# Crystal structure of the salt bis­(tri­ethano­lamine-κ^4^ *N*,*O*,*O*′,*O*′′)cadmium bis[2-(2-oxo-2,3-di­hydro-1,3-benzo­thia­zol-3-yl)acetate]

**DOI:** 10.1107/S2056989016004515

**Published:** 2016-03-22

**Authors:** Jamshid Mengnorovich Ashurov

**Affiliations:** a Institute of Bioorganic Chemistry, Academy of Sciences of Uzbekistan, M. Ulugbek Str. 83, Tashkent 700125, Uzbekistan

**Keywords:** crystal structure, cadmium(II) complex, tri­ethano­lamine, 2-(2-oxo-2,3-di­hydro-1,3-benzo­thia­zolin-3-yl)acetate, hydrogen bonding

## Abstract

The reaction of 2-(2-oxo-2,3-di­hydro-1,3-benzo­thia­zol-3-yl)acetic acid (NBTA) and tri­ethano­lamine (TEA) with Cd(CH_3_OO)_2_ resulted in the formation of the title salt, [Cd(C_6_H_15_NO_3_)_2_](C_9_H_6_NO_3_S)_2_. In its crystal structure, the complex cation [Cd(TEA)_2_]^2+^ and two independent NBTA^−^ units with essentially similar geometries and conformations are present. In the complex cation, each TEA mol­ecule behaves as an *N*,*O*,*O*′,*O*′′-tetra­dentate ligand, giving rise to an eight-coordinate Cd^II^ ion with a bicapped trigonal–prismatic configuration. All ethanol groups of each TEA mol­ecule form three five-membered chelate rings around the Cd^II^ ion. The Cd—O and Cd—N distances are in the ranges 2.392 (2)–2.478 (2) and 2.465 (2)–2.475 (3) Å, respectively. O—H⋯O hydrogen bonds between the TEA hy­droxy groups and carboxyl­ate O atoms connect cationic and anionic moieties into chains parallel to [110]. Each NBTA^−^ anion is additionally linked to a symmetry-related anion through π–π stacking inter­actions between the benzene and thia­zoline rings [minimum centroid-to-centroid separation = 3.604 (2) Å]. Together with additional C—H⋯O inter­actions, these establish a double-layer polymeric network parallel to (001).

## Abstract 2

The complex cation of the title salt, [Cd(C_6_H_15_NO_3_)_2_](C_9_H_6_NO_3_S)_2_, has a Cd^II^ atom coordinated in a bicapped trigonal–prismatic fashion by two tetra­dentate tri­ethano­lamine (TEA) ligands. The supra­molecular inter­actions between cations and anions lead to a two-dimensional network structure parallel to (001).

## Chemical context   

Tri­ethano­lamine (TEA) is a potential ligand for the incorporation of metals into metal-ion-containing supra­molecular frameworks, and many compounds constructed from TEA have been reported in the last decade (Haukka *et al.*, 2005 ▸; Topcu *et al.*, 2001 ▸; Ucar *et al.*, 2004 ▸). TEA is also used as a corrosion inhibitor in metal-cutting fluids, as a curing agent for ep­oxy and rubber polymers, adhesives, anti­static agents or as a pharmaceutical inter­mediate and an ointment emulsifier. However, TEA has no specific physiological effects (Beyer *et al.*, 1983 ▸; Knaak *et al.*, 1997 ▸), with exception of its low anti­bacterial action. Benzo­thia­zoles are bicyclic ring systems and their derivatives have been studied and found to have various chemical reactivities and biological activities. For example, benzo­thia­zole is a precursor for rubber accelerators, a component of cyanine dyes, is used as a slimicide in the paper and pulp industry, or in the production of certain fungicides, herbicides, pharmaceuticals (Bellavia *et al.*, 2000 ▸; Seo *et al.*, 2000 ▸), anti-allergic (Musser *et al.*, 1984 ▸), anti­tumor (Yoshida *et al.*, 2005 ▸) or anti-diabetic (Pattan *et al.*, 2005 ▸) substances.

The inter­action of metal ions with TEA results in the formation of complexes in which TEA demonstrates monodentate (Kumar *et al.*, 2014 ▸), bidentate (Kapteijn *et al.*, 1997 ▸; Long *et al.*, 2004 ▸), tridentate (Gao *et al.*, 2004 ▸; Ucar *et al.*, 2004 ▸; Krabbes *et al.*, 1999 ▸; Haukka *et al.*, 2005 ▸; Yeşilel *et al.*, 2004 ▸; Mirskova *et al.*, 2013 ▸) or tetra­dentate binding modes (Zaitsev *et al.*, 2014 ▸; Kazak *et al.*, 2003 ▸; Yilmaz *et al.*, 2004 ▸; Rickard *et al.*, 1999 ▸; Maestri & Brown, 2004 ▸; Kovbasyuk *et al.*, 2001 ▸; Tudor *et al.*, 2001 ▸). In some complexes, TEA has bridging properties (Langley *et al.*, 2011 ▸; Atria *et al.*, 2015 ▸; Wittick *et al.*, 2006 ▸; Sharma *et al.*, 2014 ▸; Yang *et al.*, 2014 ▸; Funes *et al.*, 2014 ▸). In addition, there are metal complexes known in which TEA mol­ecules are uncoordinating (Ilyukhin *et al.*, 2013 ▸; Manos *et al.*, 2012 ▸). As an ancillary ligand, TEA may enhance the physiological action of bioactive substances in mixed-ligand metal complexes (Boulsourani *et al.*, 2011 ▸). We have reported the synthesis of mixed-ligand complexes of Zn, Cd and Cu with TEA and *p*-nitro­benzoic acid (NBA) and determined the structures of [Cu_2_(NBA)_2_TEA](NBA)(5H_2_O), [Zn(NBA)_2_TEA] and [Cd(NBA)_2_TEA] (Ashurov *et al.*, 2015 ▸). The cobalt(II) complex [Co(C_6_H_15_NO_3_)_2_](C_9_H_6_NO_3_S)_2_, obtained by the reaction of NBTA and TEA with Co(NO_3_)_2_, has been reported (Ashurov *et al.*, 2016 ▸). Here, the synthesis and structure of the related title compound, [Cd(C_6_H_15_NO_3_)_2_](C_9_H_6_NO_3_S)_2_, (I), is described.
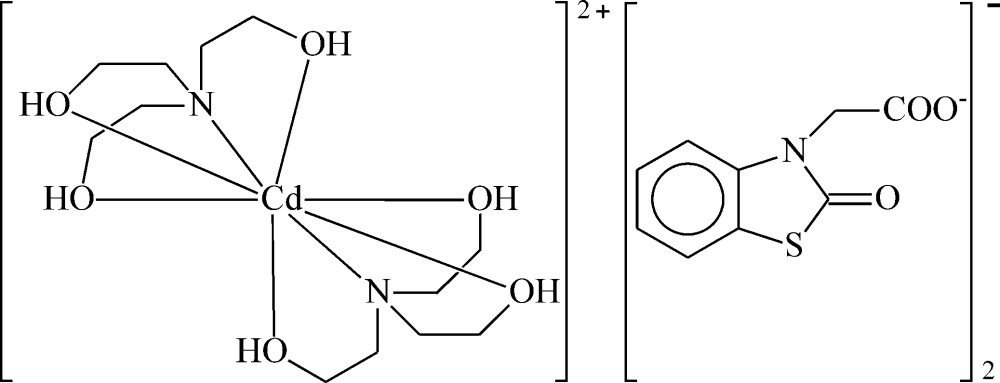


## Structural commentary   

**Figure 1:**
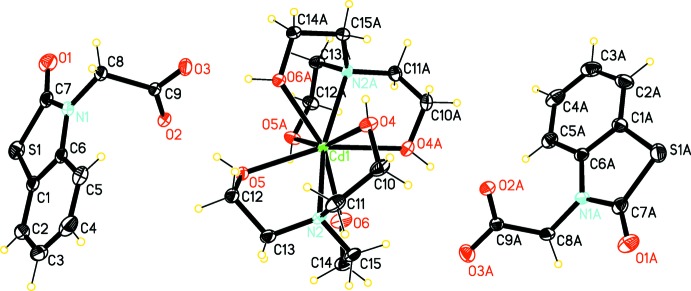
The structures of the mol­ecular moieties in the title salt. Displacement ellipsoids are drawn at the 30% probability level.

The structure of the mol­ecular entities of (I) is shown in Fig. 1 ▸; these consist of a complex cation and two independent NBTA^−^ anions. In the cationic complex, the Cd^II^ ion is ligated by two neutral TEA mol­ecules, which act as *N,O,O′*,*O*′′-tetra­dentate ligands, resulting in a bicapped trigonal–prismatic coordination polyhedron of the type CdN_2_O_6_. In the complex, Cd—O and Cd—N distances are in the range 2.392 (2)–2.478 (2) and 2.465 (2)–2.475 (3) Å, respectively. The N—Cd—O bond angles range from 68.58 (8) to 122.59 (10)° and the O—Cd—O angles are in an inter­val of 72.54 (9) to 162.13 (11)°. Both thia­zoline rings (C1/C6/N1/C7/S1 and C1*A*/C6*A*/N1*A*/C7*A*/S1*A*) and bicyclic benzo­thia­zole units (N1/S1/C1–C7 and (N1*A*/S1*A*/C1*A*–C7*A*) are close to planar, the largest deviations from the least-squares planes being 0.002 (2), 0.004 (2) and 0.008 (3), 0.005 (3) Å, respectively. The dihedral angles between the plane of the carboxyl­ate group and the attached benzo­thia­zole ring system are 77.895 (3) and 71.408 (3)° in the two anions.

## Supra­molecular features   

**Figure 2:**
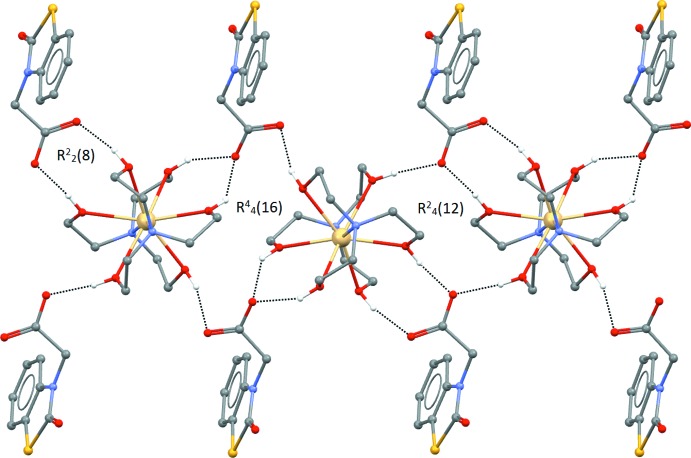
Part of the crystal structure with hydrogen bonds shown as dashed lines. For clarity, H atoms not involved in hydrogen bonding are omitted.

**Figure 3:**
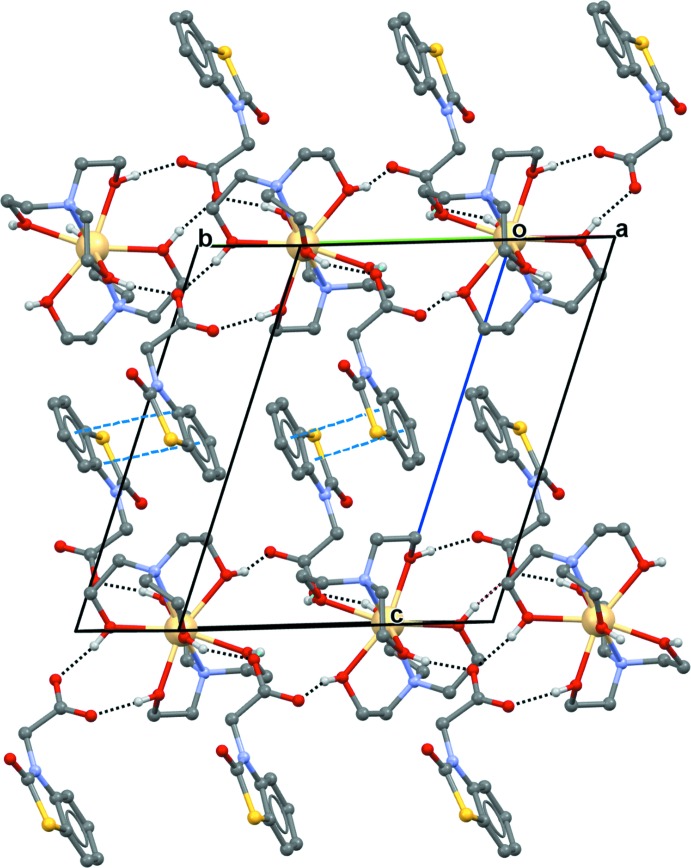
Packing of structural units in (I). Hydrogen bonds are indicated as black dashed lines and π-π stacking inter­actions as blue dashed lines.

In the crystal structure of (I), classical inter­molecular O—H⋯O hydrogen bonds are observed (Table 1 ▸) which link the complex cations and NBTA^−^ anions into a chain structure extending parallel to [110], whereby each cation is surrounded by four NBTA^−^ anions. The H atoms of all hydroxyl groups of the TEA ligands form a hydrogen bond with a carboxyl­ate O atom of the NBTA^−^ ions. In addition, there is weak hydrogen bond between one –CH_2_ group and the O1 atom of the NBTA anion, with a C⋯O distance of 3.282 (6)Å (Table 1 ▸). The above-mentioned hydrogen bonds give rise to 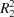
(8), 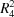
(12) and 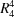
(16) graph-set motifs (Fig. 2 ▸). The NBTA^−^ anion layers are not linked by hydrogen bonds, but there are mutual π–π stacking inter­actions between benzene rings (centroid *Cg*1) and thia­zoline rings (centroid *Cg*2) of adjacent inversion-related mol­ecules [*Cg*1⋯*Cg*2 (2 − *x*, −*y*, 1 − *z*) = 3.604 (2) Å] (Fig. 3 ▸). Together, these supra­molecular inter­actions generate a double-layer polymeric network parallel to (001).

## Database survey   

A survey of the Cambridge Structural Database (CSD; Groom & Allen, 2014 ▸) showed that coordination complexes of TEA with many metals including those of the *s*-, *d*-, *p*-, and *f*-block elements have been reported. Structures containing the [Cd(TEA)_2_]^2+^ cation are deposited in the CSD with reference codes EYIPAD, MEVQIN and YOVBIU.

## Synthesis and crystallization   

To an aqueous solution (2.5 ml) of Cd(CH_3_OO)_2_ (0.103 g, 0.5 mmol) was slowly added an ethano­lic solution (5 ml) containing TEA (132 µl) and NBT (0.209 g, 1 mmol) under constant stirring. A bright-yellow crystalline product was obtained at room temperature by solvent evaporation after four weeks. Yield: 75%; calculated for C_30_H_42_CdN_4_O_12_S_2_: C, 43.56; H, 5.12; N, 6.77, found: C, 43.61; H, 5.15; N, 6.69

## Refinement details   

Crystal data, data collection and structure refinement details are summarized in Table 2 ▸. The hydroxyl H atoms of the TEA ligands were located in a difference-Fourier map and were refined with soft O—H distance restraints of 0.87 Å. The C-bound hydrogen atoms were placed in calculated positions and refined as riding atoms with C—H = 0.93 and 0.97 Å for aromatic and methyl­ene hydrogen atoms, respectively, and with *U*_iso_(H) = 1.2*U*_eq_(C).

## Supplementary Material

Crystal structure: contains datablock(s) I. DOI: 10.1107/S2056989016004515/wm5280sup1.cif

Structure factors: contains datablock(s) I. DOI: 10.1107/S2056989016004515/wm5280Isup2.hkl

CCDC reference: 1468939

Additional supporting information:  crystallographic information; 3D view; checkCIF report

## Figures and Tables

**Table 1:** Hydrogen-bond geometry (Å, °)

*D*—H⋯*A*	*D*—H	H⋯*A*	*D*⋯*A*	*D*—H⋯*A*
O4—H4⋯O3^i^	0.87 (3)	1.82 (2)	2.676 (4)	170 (4)
O4*A*—H4*A*⋯O2*A*	0.87 (3)	1.91 (3)	2.748 (4)	161 (3)
O5—H5⋯O2	0.87 (3)	1.81 (3)	2.661 (3)	166 (3)
O5*A*—H5*A*⋯O3*A* ^ii^	0.87 (2)	1.83 (3)	2.640 (4)	154 (2)
O6—H6⋯O3*A* ^ii^	0.85 (2)	2.04 (2)	2.749 (5)	141 (3)
O6*A*—H6*A*⋯O3	0.86 (2)	1.79 (2)	2.640 (4)	172 (2)
C11*A*—H11*D*⋯O1^iii^	0.97	2.47	3.282 (6)	142

Symmetry codes: (i) 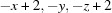; (ii) 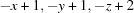; (iii) 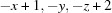.

**Table 2:** Experimental details

Crystal data	
Chemical formula	[Cd(C_6_H_15_NO_3_)_2_](C_9_H_6_NO_3_S)_2_
*M*_r_	827.20
Crystal system, space group	Triclinic, *P* 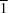
Temperature (K)	293
*a*, *b*, *c* (Å)	10.7061 (5), 12.2157 (5), 14.6159 (8)
α, β, γ (°)	65.520 (5), 79.600 (4), 82.417 (4)
*V* (Å^3^)	1707.59 (14)
*Z*	2
Radiation type	Cu *K*α
μ (mm^−1^)	6.85
Crystal size (mm)	0.6 × 0.3 × 0.2

Data collection	
Diffractometer	Oxford Diffraction Xcalibur Ruby
Absorption correction	Multi-scan (*CrysAlis PRO*; Oxford Diffraction, 2009 ▸)
*T*_min_, *T*_max_	0.127, 0.254
No. of measured, independent and observed [*I* > 2σ(*I*)] reflections	14752, 7001, 6533
*R*_int_	0.047
(sin θ/λ)_max_ (Å^−1^)	0.629

Refinement	
*R*[*F* ^2^ > 2σ(*F* ^2^)], *wR*(*F* ^2^), *S*	0.040, 0.107, 1.06
No. of reflections	7001
No. of parameters	461
No. of restraints	18
H-atom treatment	H atoms treated by a mixture of independent and constrained refinement
Δρ_max_, Δρ_min_ (e Å^−3^)	1.20, −0.67

Computer programs: *CrysAlis PRO* (Oxford Diffraction, 2009 ▸), *SHELXS97*, *SHELXL97* and *XP* in *SHELXTL* (Sheldrick, 2008 ▸) and *Mercury* (Macrae *et al.*, 2006 ▸).

## supplementary crystallographic information

### Crystal data

**Table:**

[Cd(C_6_H_15_NO_3_)_2_](C_9_H_6_NO_3_S)_2_	*Z* = 2
*M**_r_* = 827.20	*F*(000) = 852
Triclinic, *P*1	*D*_x_ = 1.609 Mg m^−^^3^
Hall symbol: -P 1	Cu *K*α radiation, λ = 1.54184 Å
*a* = 10.7061 (5) Å	Cell parameters from 10769 reflections
*b* = 12.2157 (5) Å	θ = 3.4–75.7°
*c* = 14.6159 (8) Å	µ = 6.85 mm^−^^1^
α = 65.520 (5)°	*T* = 293 K
β = 79.600 (4)°	Block, bright yellow
γ = 82.417 (4)°	0.6 × 0.3 × 0.2 mm
*V* = 1707.59 (14) Å^3^	

### Data collection

**Table:**

Oxford Diffraction Xcalibur Ruby diffractometer	7001 independent reflections
Radiation source: fine-focus sealed tube	6533 reflections with *I* > 2σ(*I*)
Graphite monochromator	*R*_int_ = 0.047
Detector resolution: 10.2576 pixels mm^-1^	θ_max_ = 75.9°, θ_min_ = 3.4°
ω scans	*h* = −13→12
Absorption correction: multi-scan (*CrysAlis PRO*; Oxford Diffraction, 2009)	*k* = −14→15
*T*_min_ = 0.127, *T*_max_ = 0.254	*l* = −17→18
14752 measured reflections	

### Refinement

**Table:**

Refinement on *F*^2^	Secondary atom site location: difference Fourier map
Least-squares matrix: full	Hydrogen site location: inferred from neighbouring sites
*R*[*F*^2^ > 2σ(*F*^2^)] = 0.040	H atoms treated by a mixture of independent and constrained refinement
*wR*(*F*^2^) = 0.107	*w* = 1/[σ^2^(*F*_o_^2^) + (0.0692*P*)^2^ + 0.3219*P*] where *P* = (*F*_o_^2^ + 2*F*_c_^2^)/3
*S* = 1.06	(Δ/σ)_max_ = 0.001
7001 reflections	Δρ_max_ = 1.20 e Å^−^^3^
461 parameters	Δρ_min_ = −0.67 e Å^−^^3^
18 restraints	Extinction correction: *SHELXL97* (Sheldrick, 2008), Fc^*^=kFc[1+0.001xFc^2^λ^3^/sin(2θ)]^-1/4^
Primary atom site location: structure-invariant direct methods	Extinction coefficient: 0.00078 (13)

### Special details

**Table:**

Geometry. All e.s.d.'s (except the e.s.d. in the dihedral angle between two l.s. planes) are estimated using the full covariance matrix. The cell e.s.d.'s are taken into account individually in the estimation of e.s.d.'s in distances, angles and torsion angles; correlations between e.s.d.'s in cell parameters are only used when they are defined by crystal symmetry. An approximate (isotropic) treatment of cell e.s.d.'s is used for estimating e.s.d.'s involving l.s. planes.
Refinement. Refinement of *F*^2^ against ALL reflections. The weighted *R*-factor *wR* and goodness of fit *S* are based on *F*^2^, conventional *R*-factors *R* are based on *F*, with *F* set to zero for negative *F*^2^. The threshold expression of *F*^2^ > σ(*F*^2^) is used only for calculating *R*-factors(gt) *etc*. and is not relevant to the choice of reflections for refinement. *R*-factors based on *F*^2^ are statistically about twice as large as those based on *F*, and *R*- factors based on ALL data will be even larger.

### Fractional atomic coordinates and isotropic or equivalent isotropic displacement parameters (Å^2^)

**Table:**

	*x*	*y*	*z*	*U*_iso_*/*U*_eq_	
Cd1	0.712574 (17)	0.241527 (14)	0.997601 (13)	0.02746 (9)	
O6A	0.8300 (3)	0.0513 (2)	1.00868 (17)	0.0434 (5)	
H6A	0.855 (4)	0.0269 (19)	0.9608 (12)	0.065*	
O5A	0.5116 (2)	0.1940 (2)	0.97875 (18)	0.0444 (5)	
H5A	0.4541 (16)	0.247 (2)	0.947 (2)	0.067*	
O5	0.7418 (2)	0.24576 (19)	0.82842 (16)	0.0368 (5)	
H5	0.736 (3)	0.186 (2)	0.8118 (19)	0.055*	
O4	0.9043 (2)	0.2228 (2)	1.0772 (2)	0.0457 (6)	
H4	0.960 (2)	0.1633 (14)	1.100 (3)	0.069*	
O4A	0.6459 (3)	0.3096 (2)	1.13739 (19)	0.0462 (6)	
H4A	0.668 (4)	0.3705 (16)	1.1455 (16)	0.069*	
N2A	0.6329 (2)	0.0713 (2)	1.15330 (18)	0.0310 (5)	
N2	0.8509 (2)	0.4053 (2)	0.8822 (2)	0.0333 (5)	
O6	0.5915 (3)	0.4392 (3)	0.9324 (3)	0.0594 (7)	
H6	0.5116 (9)	0.453 (2)	0.934 (4)	0.089*	
C13A	0.5318 (3)	0.0196 (3)	1.1293 (3)	0.0405 (7)	
H13A	0.4851	−0.0337	1.1921	0.049*	
H13B	0.5707	−0.0279	1.0912	0.049*	
C15A	0.7417 (3)	−0.0170 (3)	1.1835 (2)	0.0383 (7)	
H15A	0.7114	−0.0886	1.2408	0.046*	
H15B	0.8014	0.0170	1.2056	0.046*	
C14	0.6569 (4)	0.5438 (3)	0.8689 (3)	0.0474 (8)	
H14A	0.6188	0.6117	0.8845	0.057*	
H14B	0.6518	0.5628	0.7983	0.057*	
C12A	0.4410 (3)	0.1157 (3)	1.0688 (3)	0.0431 (7)	
H12A	0.3780	0.0794	1.0521	0.052*	
H12B	0.3973	0.1604	1.1079	0.052*	
C14A	0.8105 (3)	−0.0530 (3)	1.0995 (2)	0.0404 (7)	
H14C	0.8918	−0.0948	1.1175	0.048*	
H14D	0.7605	−0.1072	1.0900	0.048*	
C15	0.7929 (3)	0.5198 (3)	0.8870 (3)	0.0430 (7)	
H15C	0.8415	0.5856	0.8366	0.052*	
H15D	0.7979	0.5178	0.9533	0.052*	
C13	0.8646 (4)	0.4119 (3)	0.7768 (3)	0.0445 (8)	
H13C	0.9443	0.4465	0.7394	0.053*	
H13D	0.7960	0.4645	0.7429	0.053*	
C10	0.9712 (4)	0.3297 (3)	1.0288 (3)	0.0514 (9)	
H10A	1.0567	0.3125	1.0467	0.062*	
H10B	0.9289	0.3898	1.0531	0.062*	
C11	0.9783 (3)	0.3794 (3)	0.9157 (3)	0.0501 (9)	
H11A	1.0229	0.4531	0.8859	0.060*	
H11B	1.0270	0.3219	0.8909	0.060*	
C12	0.8618 (3)	0.2906 (3)	0.7759 (3)	0.0405 (7)	
H12C	0.8722	0.2968	0.7066	0.049*	
H12D	0.9302	0.2369	0.8094	0.049*	
C11A	0.5800 (4)	0.1123 (3)	1.2352 (3)	0.0456 (8)	
H11C	0.5818	0.0443	1.3002	0.055*	
H11D	0.4918	0.1409	1.2286	0.055*	
C10A	0.6504 (4)	0.2095 (3)	1.2338 (3)	0.0480 (8)	
H10C	0.6118	0.2341	1.2883	0.058*	
H10D	0.7381	0.1812	1.2433	0.058*	
S1	0.71637 (9)	0.01677 (10)	0.49722 (8)	0.0535 (2)	
O2	0.7640 (2)	0.05730 (19)	0.77831 (17)	0.0375 (5)	
O3	0.9143 (3)	−0.0435 (2)	0.87446 (19)	0.0475 (6)	
N1	0.8544 (3)	−0.0361 (2)	0.6378 (2)	0.0384 (6)	
O1	0.7093 (3)	−0.1763 (3)	0.6719 (3)	0.0660 (8)	
C6	0.8954 (3)	0.0728 (3)	0.5643 (2)	0.0362 (6)	
C1	0.8305 (3)	0.1163 (3)	0.4801 (3)	0.0408 (7)	
C9	0.8546 (3)	−0.0180 (3)	0.8009 (2)	0.0333 (6)	
C8	0.8983 (4)	−0.0917 (3)	0.7365 (3)	0.0463 (8)	
H8A	0.9906	−0.1007	0.7269	0.056*	
H8B	0.8667	−0.1715	0.7727	0.056*	
C7	0.7584 (3)	−0.0826 (3)	0.6175 (3)	0.0438 (8)	
C5	0.9898 (4)	0.1373 (4)	0.5677 (3)	0.0493 (8)	
H5B	1.0324	0.1099	0.6240	0.059*	
C4	1.0196 (4)	0.2437 (4)	0.4851 (4)	0.0646 (12)	
H4B	1.0839	0.2877	0.4856	0.078*	
C2	0.8590 (5)	0.2232 (4)	0.3992 (3)	0.0555 (10)	
H2	0.8145	0.2527	0.3438	0.067*	
C3	0.9553 (5)	0.2852 (4)	0.4026 (4)	0.0656 (12)	
H3	0.9772	0.3567	0.3478	0.079*	
S1A	0.79635 (10)	0.49886 (10)	1.49702 (8)	0.0570 (2)	
N1A	0.6853 (3)	0.5582 (2)	1.3394 (2)	0.0411 (6)	
O2A	0.7500 (3)	0.4620 (2)	1.19404 (19)	0.0455 (5)	
C2A	0.6322 (5)	0.3091 (4)	1.5830 (3)	0.0633 (12)	
H2A	0.6654	0.2787	1.6440	0.076*	
O3A	0.6440 (3)	0.6114 (3)	1.0843 (2)	0.0602 (7)	
O1A	0.8392 (3)	0.6849 (3)	1.3194 (3)	0.0691 (8)	
C6A	0.6268 (3)	0.4562 (3)	1.4111 (2)	0.0378 (7)	
C7A	0.7774 (4)	0.5974 (3)	1.3696 (3)	0.0474 (8)	
C8A	0.6497 (4)	0.6233 (3)	1.2389 (3)	0.0456 (8)	
H8AA	0.6891	0.6998	1.2081	0.055*	
H8AB	0.5583	0.6406	1.2455	0.055*	
C1A	0.6771 (4)	0.4107 (3)	1.5032 (3)	0.0456 (8)	
C5A	0.5312 (4)	0.4003 (4)	1.3989 (3)	0.0508 (9)	
H5AA	0.4984	0.4301	1.3378	0.061*	
C9A	0.6860 (3)	0.5590 (3)	1.1673 (2)	0.0353 (6)	
C3A	0.5362 (5)	0.2531 (4)	1.5704 (4)	0.0703 (14)	
H3A	0.5055	0.1838	1.6234	0.084*	
C4A	0.4852 (4)	0.2984 (4)	1.4801 (4)	0.0645 (12)	
H4AA	0.4195	0.2603	1.4739	0.077*	

### Atomic displacement parameters (Å^2^)

**Table:**

	*U*^11^	*U*^22^	*U*^33^	*U*^12^	*U*^13^	*U*^23^
Cd1	0.03033 (13)	0.02172 (12)	0.02883 (12)	−0.00431 (7)	−0.00164 (8)	−0.00887 (8)
O6A	0.0632 (15)	0.0304 (11)	0.0320 (11)	0.0038 (10)	0.0008 (10)	−0.0131 (9)
O5A	0.0400 (12)	0.0439 (13)	0.0370 (11)	−0.0092 (10)	−0.0066 (10)	−0.0017 (10)
O5	0.0450 (12)	0.0319 (10)	0.0331 (10)	−0.0079 (9)	0.0024 (9)	−0.0143 (9)
O4	0.0386 (12)	0.0331 (11)	0.0648 (16)	−0.0003 (9)	−0.0148 (11)	−0.0165 (11)
O4A	0.0587 (15)	0.0375 (12)	0.0485 (13)	0.0007 (10)	−0.0069 (11)	−0.0247 (11)
N2A	0.0339 (12)	0.0293 (11)	0.0300 (11)	−0.0041 (9)	−0.0015 (10)	−0.0125 (10)
N2	0.0343 (13)	0.0246 (11)	0.0431 (14)	−0.0049 (9)	−0.0023 (10)	−0.0159 (10)
O6	0.0388 (13)	0.0431 (14)	0.085 (2)	−0.0025 (11)	−0.0041 (14)	−0.0170 (14)
C13A	0.0410 (17)	0.0305 (14)	0.0459 (17)	−0.0117 (12)	−0.0052 (14)	−0.0089 (13)
C15A	0.0471 (18)	0.0315 (14)	0.0336 (15)	0.0026 (12)	−0.0101 (13)	−0.0100 (12)
C14	0.050 (2)	0.0336 (16)	0.053 (2)	0.0060 (14)	−0.0059 (16)	−0.0144 (15)
C12A	0.0325 (15)	0.0476 (18)	0.0395 (16)	−0.0104 (13)	−0.0009 (13)	−0.0072 (15)
C14A	0.0499 (18)	0.0283 (14)	0.0394 (16)	0.0076 (13)	−0.0077 (14)	−0.0123 (13)
C15	0.0494 (19)	0.0256 (14)	0.0540 (19)	−0.0066 (12)	−0.0001 (15)	−0.0176 (14)
C13	0.057 (2)	0.0291 (15)	0.0405 (17)	−0.0121 (13)	0.0104 (15)	−0.0116 (13)
C10	0.0445 (19)	0.0366 (17)	0.080 (3)	−0.0048 (14)	−0.0231 (18)	−0.0240 (18)
C11	0.0341 (17)	0.0399 (18)	0.079 (3)	−0.0053 (13)	−0.0051 (17)	−0.0273 (18)
C12	0.0480 (18)	0.0344 (15)	0.0383 (16)	−0.0075 (13)	0.0055 (14)	−0.0171 (13)
C11A	0.054 (2)	0.0467 (19)	0.0336 (16)	−0.0024 (15)	0.0053 (14)	−0.0181 (14)
C10A	0.061 (2)	0.051 (2)	0.0403 (17)	−0.0004 (16)	−0.0052 (16)	−0.0282 (16)
S1	0.0509 (5)	0.0601 (5)	0.0625 (6)	0.0047 (4)	−0.0210 (4)	−0.0347 (5)
O2	0.0419 (12)	0.0328 (10)	0.0408 (11)	0.0018 (9)	−0.0047 (9)	−0.0193 (9)
O3	0.0560 (15)	0.0518 (14)	0.0432 (13)	0.0144 (11)	−0.0160 (11)	−0.0289 (12)
N1	0.0504 (16)	0.0363 (13)	0.0308 (12)	0.0019 (11)	−0.0050 (11)	−0.0173 (11)
O1	0.0657 (18)	0.0528 (17)	0.075 (2)	−0.0178 (14)	0.0162 (16)	−0.0277 (15)
C6	0.0397 (16)	0.0364 (15)	0.0368 (15)	0.0057 (12)	−0.0037 (13)	−0.0219 (13)
C1	0.0475 (18)	0.0391 (16)	0.0410 (16)	0.0087 (13)	−0.0073 (14)	−0.0240 (14)
C9	0.0407 (16)	0.0286 (13)	0.0306 (14)	−0.0007 (11)	−0.0009 (12)	−0.0139 (11)
C8	0.067 (2)	0.0382 (17)	0.0354 (16)	0.0153 (15)	−0.0129 (16)	−0.0194 (14)
C7	0.0441 (18)	0.0454 (18)	0.0467 (18)	−0.0006 (14)	0.0033 (14)	−0.0277 (16)
C5	0.0456 (19)	0.052 (2)	0.062 (2)	0.0000 (15)	−0.0057 (17)	−0.0362 (19)
C4	0.060 (2)	0.055 (2)	0.089 (3)	−0.0164 (19)	0.013 (2)	−0.044 (2)
C2	0.077 (3)	0.0425 (19)	0.0419 (19)	0.0118 (18)	−0.0065 (18)	−0.0170 (16)
C3	0.090 (3)	0.0377 (19)	0.058 (2)	−0.005 (2)	0.015 (2)	−0.0182 (18)
S1A	0.0626 (6)	0.0600 (6)	0.0567 (5)	0.0088 (4)	−0.0195 (5)	−0.0307 (5)
N1A	0.0558 (17)	0.0300 (13)	0.0342 (13)	−0.0024 (11)	−0.0031 (12)	−0.0109 (11)
O2A	0.0572 (15)	0.0322 (11)	0.0461 (13)	0.0045 (10)	−0.0083 (11)	−0.0164 (10)
C2A	0.087 (3)	0.048 (2)	0.0381 (19)	0.008 (2)	0.002 (2)	−0.0083 (17)
O3A	0.0721 (19)	0.0615 (17)	0.0503 (15)	0.0130 (14)	−0.0267 (14)	−0.0235 (14)
O1A	0.0694 (19)	0.0522 (17)	0.085 (2)	−0.0193 (14)	0.0006 (17)	−0.0263 (16)
C6A	0.0432 (17)	0.0291 (14)	0.0382 (15)	0.0040 (12)	0.0009 (13)	−0.0152 (13)
C7A	0.0486 (19)	0.0440 (18)	0.053 (2)	−0.0005 (15)	−0.0009 (16)	−0.0268 (17)
C8A	0.065 (2)	0.0266 (14)	0.0405 (17)	0.0064 (14)	−0.0084 (16)	−0.0107 (13)
C1A	0.055 (2)	0.0383 (17)	0.0373 (16)	0.0094 (15)	−0.0017 (15)	−0.0150 (14)
C5A	0.050 (2)	0.0458 (19)	0.058 (2)	−0.0030 (15)	0.0010 (17)	−0.0256 (18)
C9A	0.0370 (16)	0.0314 (14)	0.0358 (15)	−0.0042 (11)	−0.0051 (12)	−0.0114 (12)
C3A	0.087 (3)	0.044 (2)	0.057 (3)	−0.008 (2)	0.023 (2)	−0.0100 (19)
C4A	0.059 (2)	0.048 (2)	0.084 (3)	−0.0146 (18)	0.019 (2)	−0.033 (2)

### Geometric parameters (Å, º)

**Table:**

Cd1—O5A	2.392 (2)	C12—H12C	0.9700
Cd1—O5	2.415 (2)	C12—H12D	0.9700
Cd1—O6A	2.449 (2)	C11A—C10A	1.481 (5)
Cd1—N2A	2.465 (2)	C11A—H11C	0.9700
Cd1—O4A	2.470 (2)	C11A—H11D	0.9700
Cd1—N2	2.475 (3)	C10A—H10C	0.9700
Cd1—O6	2.476 (3)	C10A—H10D	0.9700
Cd1—O4	2.478 (2)	S1—C1	1.750 (4)
O6A—C14A	1.413 (4)	S1—C7	1.772 (4)
O6A—H6A	0.856 (9)	O2—C9	1.234 (4)
O5A—C12A	1.416 (4)	O3—C9	1.256 (4)
O5A—H5A	0.871 (10)	N1—C7	1.368 (5)
O5—C12	1.422 (4)	N1—C6	1.384 (4)
O5—H5	0.872 (10)	N1—C8	1.453 (4)
O4—C10	1.420 (4)	O1—C7	1.213 (5)
O4—H4	0.866 (9)	C6—C5	1.381 (5)
O4A—C10A	1.436 (5)	C6—C1	1.396 (5)
O4A—H4A	0.870 (10)	C1—C2	1.375 (5)
N2A—C15A	1.475 (4)	C9—C8	1.533 (4)
N2A—C11A	1.477 (4)	C8—H8A	0.9700
N2A—C13A	1.480 (4)	C8—H8B	0.9700
N2—C15	1.478 (4)	C5—C4	1.385 (6)
N2—C11	1.482 (4)	C5—H5B	0.9300
N2—C13	1.490 (4)	C4—C3	1.374 (7)
O6—C14	1.415 (5)	C4—H4B	0.9300
O6—H6	0.848 (9)	C2—C3	1.376 (7)
C13A—C12A	1.502 (5)	C2—H2	0.9300
C13A—H13A	0.9700	C3—H3	0.9300
C13A—H13B	0.9700	S1A—C1A	1.743 (4)
C15A—C14A	1.509 (4)	S1A—C7A	1.781 (4)
C15A—H15A	0.9700	N1A—C7A	1.362 (5)
C15A—H15B	0.9700	N1A—C6A	1.389 (4)
C14—C15	1.495 (5)	N1A—C8A	1.446 (4)
C14—H14A	0.9700	O2A—C9A	1.235 (4)
C14—H14B	0.9700	C2A—C1A	1.373 (6)
C12A—H12A	0.9700	C2A—C3A	1.384 (8)
C12A—H12B	0.9700	C2A—H2A	0.9300
C14A—H14C	0.9700	O3A—C9A	1.249 (4)
C14A—H14D	0.9700	O1A—C7A	1.216 (5)
C15—H15C	0.9700	C6A—C5A	1.376 (5)
C15—H15D	0.9700	C6A—C1A	1.406 (5)
C13—C12	1.492 (4)	C8A—C9A	1.522 (4)
C13—H13C	0.9700	C8A—H8AA	0.9700
C13—H13D	0.9700	C8A—H8AB	0.9700
C10—C11	1.498 (6)	C5A—C4A	1.388 (6)
C10—H10A	0.9700	C5A—H5AA	0.9300
C10—H10B	0.9700	C3A—C4A	1.386 (8)
C11—H11A	0.9700	C3A—H3A	0.9300
C11—H11B	0.9700	C4A—H4AA	0.9300
			
O5A—Cd1—O5	75.23 (8)	H13C—C13—H13D	108.0
O5A—Cd1—O6A	97.23 (9)	O4—C10—C11	111.8 (3)
O5—Cd1—O6A	74.12 (7)	O4—C10—H10A	109.2
O5A—Cd1—N2A	70.99 (8)	C11—C10—H10A	109.2
O5—Cd1—N2A	124.88 (8)	O4—C10—H10B	109.2
O6A—Cd1—N2A	68.58 (8)	C11—C10—H10B	109.2
O5A—Cd1—O4A	100.09 (9)	H10A—C10—H10B	107.9
O5—Cd1—O4A	159.22 (8)	N2—C11—C10	112.5 (3)
O6A—Cd1—O4A	126.65 (8)	N2—C11—H11A	109.1
N2A—Cd1—O4A	70.34 (8)	C10—C11—H11A	109.1
O5A—Cd1—N2	130.03 (8)	N2—C11—H11B	109.1
O5—Cd1—N2	70.57 (8)	C10—C11—H11B	109.1
O6A—Cd1—N2	106.90 (8)	H11A—C11—H11B	107.8
N2A—Cd1—N2	158.75 (9)	O5—C12—C13	107.1 (3)
O4A—Cd1—N2	99.33 (9)	O5—C12—H12C	110.3
O5A—Cd1—O6	75.81 (9)	C13—C12—H12C	110.3
O5—Cd1—O6	88.12 (10)	O5—C12—H12D	110.3
O6A—Cd1—O6	162.13 (10)	C13—C12—H12D	110.3
N2A—Cd1—O6	122.59 (9)	H12C—C12—H12D	108.6
O4A—Cd1—O6	71.14 (10)	N2A—C11A—C10A	113.0 (3)
N2—Cd1—O6	67.86 (9)	N2A—C11A—H11C	109.0
O5A—Cd1—O4	158.32 (8)	C10A—C11A—H11C	109.0
O5—Cd1—O4	118.25 (9)	N2A—C11A—H11D	109.0
O6A—Cd1—O4	72.54 (9)	C10A—C11A—H11D	109.0
N2A—Cd1—O4	87.39 (8)	H11C—C11A—H11D	107.8
O4A—Cd1—O4	73.06 (9)	O4A—C10A—C11A	108.1 (3)
N2—Cd1—O4	71.65 (9)	O4A—C10A—H10C	110.1
O6—Cd1—O4	119.25 (9)	C11A—C10A—H10C	110.1
C14A—O6A—Cd1	119.37 (18)	O4A—C10A—H10D	110.1
C14A—O6A—H6A	106.6 (15)	C11A—C10A—H10D	110.1
Cd1—O6A—H6A	127.9 (16)	H10C—C10A—H10D	108.4
C12A—O5A—Cd1	115.7 (2)	C1—S1—C7	92.04 (17)
C12A—O5A—H5A	103.9 (15)	C7—N1—C6	116.2 (3)
Cd1—O5A—H5A	124.9 (17)	C7—N1—C8	120.8 (3)
C12—O5—Cd1	109.85 (18)	C6—N1—C8	122.6 (3)
C12—O5—H5	103.7 (15)	C5—C6—N1	126.8 (3)
Cd1—O5—H5	127.1 (17)	C5—C6—C1	120.1 (3)
C10—O4—Cd1	111.1 (2)	N1—C6—C1	113.0 (3)
C10—O4—H4	107.2 (16)	C2—C1—C6	121.1 (4)
Cd1—O4—H4	131.4 (17)	C2—C1—S1	128.9 (3)
C10A—O4A—Cd1	110.41 (18)	C6—C1—S1	110.0 (3)
C10A—O4A—H4A	105.2 (16)	O2—C9—O3	126.3 (3)
Cd1—O4A—H4A	130.4 (18)	O2—C9—C8	118.3 (3)
C15A—N2A—C11A	110.2 (3)	O3—C9—C8	115.4 (3)
C15A—N2A—C13A	111.4 (2)	N1—C8—C9	112.6 (3)
C11A—N2A—C13A	109.5 (3)	N1—C8—H8A	109.1
C15A—N2A—Cd1	107.07 (18)	C9—C8—H8A	109.1
C11A—N2A—Cd1	110.73 (19)	N1—C8—H8B	109.1
C13A—N2A—Cd1	107.92 (18)	C9—C8—H8B	109.1
C15—N2—C11	109.8 (3)	H8A—C8—H8B	107.8
C15—N2—C13	110.8 (3)	O1—C7—N1	126.4 (4)
C11—N2—C13	109.0 (3)	O1—C7—S1	124.9 (3)
C15—N2—Cd1	108.45 (19)	N1—C7—S1	108.7 (3)
C11—N2—Cd1	109.2 (2)	C6—C5—C4	118.4 (4)
C13—N2—Cd1	109.68 (18)	C6—C5—H5B	120.8
C14—O6—Cd1	119.6 (2)	C4—C5—H5B	120.8
C14—O6—H6	111.6 (15)	C3—C4—C5	120.8 (4)
Cd1—O6—H6	127.8 (15)	C3—C4—H4B	119.6
N2A—C13A—C12A	111.9 (3)	C5—C4—H4B	119.6
N2A—C13A—H13A	109.2	C1—C2—C3	118.1 (4)
C12A—C13A—H13A	109.2	C1—C2—H2	120.9
N2A—C13A—H13B	109.2	C3—C2—H2	120.9
C12A—C13A—H13B	109.2	C4—C3—C2	121.4 (4)
H13A—C13A—H13B	107.9	C4—C3—H3	119.3
N2A—C15A—C14A	113.5 (3)	C2—C3—H3	119.3
N2A—C15A—H15A	108.9	C1A—S1A—C7A	91.44 (18)
C14A—C15A—H15A	108.9	C7A—N1A—C6A	116.3 (3)
N2A—C15A—H15B	108.9	C7A—N1A—C8A	120.6 (3)
C14A—C15A—H15B	108.9	C6A—N1A—C8A	123.1 (3)
H15A—C15A—H15B	107.7	C1A—C2A—C3A	118.4 (4)
O6—C14—C15	108.2 (3)	C1A—C2A—H2A	120.8
O6—C14—H14A	110.1	C3A—C2A—H2A	120.8
C15—C14—H14A	110.1	C5A—C6A—N1A	127.0 (3)
O6—C14—H14B	110.1	C5A—C6A—C1A	120.8 (3)
C15—C14—H14B	110.1	N1A—C6A—C1A	112.2 (3)
H14A—C14—H14B	108.4	O1A—C7A—N1A	126.9 (4)
O5A—C12A—C13A	108.3 (3)	O1A—C7A—S1A	123.9 (3)
O5A—C12A—H12A	110.0	N1A—C7A—S1A	109.2 (3)
C13A—C12A—H12A	110.0	N1A—C8A—C9A	115.5 (3)
O5A—C12A—H12B	110.0	N1A—C8A—H8AA	108.4
C13A—C12A—H12B	110.0	C9A—C8A—H8AA	108.4
H12A—C12A—H12B	108.4	N1A—C8A—H8AB	108.4
O6A—C14A—C15A	109.3 (2)	C9A—C8A—H8AB	108.4
O6A—C14A—H14C	109.8	H8AA—C8A—H8AB	107.5
C15A—C14A—H14C	109.8	C2A—C1A—C6A	120.6 (4)
O6A—C14A—H14D	109.8	C2A—C1A—S1A	128.5 (3)
C15A—C14A—H14D	109.8	C6A—C1A—S1A	110.9 (3)
H14C—C14A—H14D	108.3	C6A—C5A—C4A	118.4 (4)
N2—C15—C14	113.1 (3)	C6A—C5A—H5AA	120.8
N2—C15—H15C	108.9	C4A—C5A—H5AA	120.8
C14—C15—H15C	108.9	O2A—C9A—O3A	125.5 (3)
N2—C15—H15D	108.9	O2A—C9A—C8A	120.0 (3)
C14—C15—H15D	108.9	O3A—C9A—C8A	114.5 (3)
H15C—C15—H15D	107.8	C2A—C3A—C4A	121.2 (4)
N2—C13—C12	111.6 (3)	C2A—C3A—H3A	119.4
N2—C13—H13C	109.3	C4A—C3A—H3A	119.4
C12—C13—H13C	109.3	C3A—C4A—C5A	120.6 (5)
N2—C13—H13D	109.3	C3A—C4A—H4AA	119.7
C12—C13—H13D	109.3	C5A—C4A—H4AA	119.7
			
O5A—Cd1—O6A—C14A	−75.5 (3)	C15A—N2A—C13A—C12A	−162.3 (3)
O5—Cd1—O6A—C14A	−147.9 (3)	C11A—N2A—C13A—C12A	75.6 (3)
N2A—Cd1—O6A—C14A	−9.3 (2)	Cd1—N2A—C13A—C12A	−45.0 (3)
O4A—Cd1—O6A—C14A	32.8 (3)	C11A—N2A—C15A—C14A	−173.5 (3)
N2—Cd1—O6A—C14A	148.7 (2)	C13A—N2A—C15A—C14A	64.7 (4)
O6—Cd1—O6A—C14A	−141.2 (3)	Cd1—N2A—C15A—C14A	−53.0 (3)
O4—Cd1—O6A—C14A	84.9 (3)	Cd1—O6—C14—C15	−22.1 (4)
O5—Cd1—O5A—C12A	148.5 (2)	Cd1—O5A—C12A—C13A	−40.0 (4)
O6A—Cd1—O5A—C12A	77.1 (2)	N2A—C13A—C12A—O5A	57.6 (4)
N2A—Cd1—O5A—C12A	12.7 (2)	Cd1—O6A—C14A—C15A	−14.3 (4)
O4A—Cd1—O5A—C12A	−52.3 (2)	N2A—C15A—C14A—O6A	45.6 (4)
N2—Cd1—O5A—C12A	−163.6 (2)	C11—N2—C15—C14	−171.7 (3)
O6—Cd1—O5A—C12A	−119.7 (3)	C13—N2—C15—C14	67.9 (4)
O4—Cd1—O5A—C12A	17.0 (4)	Cd1—N2—C15—C14	−52.5 (3)
O5A—Cd1—O5—C12	172.1 (2)	O6—C14—C15—N2	49.7 (4)
O6A—Cd1—O5—C12	−85.8 (2)	C15—N2—C13—C12	−152.6 (3)
N2A—Cd1—O5—C12	−134.39 (19)	C11—N2—C13—C12	86.5 (3)
O4A—Cd1—O5—C12	92.7 (3)	Cd1—N2—C13—C12	−32.9 (3)
N2—Cd1—O5—C12	29.11 (19)	Cd1—O4—C10—C11	−42.0 (3)
O6—Cd1—O5—C12	96.3 (2)	C15—N2—C11—C10	78.3 (3)
O4—Cd1—O5—C12	−26.2 (2)	C13—N2—C11—C10	−160.2 (3)
O5A—Cd1—O4—C10	−165.5 (3)	Cd1—N2—C11—C10	−40.4 (3)
O5—Cd1—O4—C10	69.8 (2)	O4—C10—C11—N2	57.5 (4)
O6A—Cd1—O4—C10	130.2 (3)	Cd1—O5—C12—C13	−56.8 (3)
N2A—Cd1—O4—C10	−161.5 (2)	N2—C13—C12—O5	60.9 (4)
O4A—Cd1—O4—C10	−91.2 (2)	C15A—N2A—C11A—C10A	82.7 (4)
N2—Cd1—O4—C10	15.0 (2)	C13A—N2A—C11A—C10A	−154.5 (3)
O6—Cd1—O4—C10	−35.1 (3)	Cd1—N2A—C11A—C10A	−35.6 (4)
O5A—Cd1—O4A—C10A	89.6 (2)	Cd1—O4A—C10A—C11A	−50.9 (3)
O5—Cd1—O4A—C10A	164.5 (2)	N2A—C11A—C10A—O4A	59.1 (4)
O6A—Cd1—O4A—C10A	−17.3 (3)	C7—N1—C6—C5	179.6 (3)
N2A—Cd1—O4A—C10A	24.1 (2)	C8—N1—C6—C5	7.1 (5)
N2—Cd1—O4A—C10A	−136.6 (2)	C7—N1—C6—C1	−0.5 (4)
O6—Cd1—O4A—C10A	160.7 (3)	C8—N1—C6—C1	−173.1 (3)
O4—Cd1—O4A—C10A	−69.2 (2)	C5—C6—C1—C2	0.2 (5)
O5A—Cd1—N2A—C15A	137.1 (2)	N1—C6—C1—C2	−179.7 (3)
O5—Cd1—N2A—C15A	81.8 (2)	C5—C6—C1—S1	−179.6 (2)
O6A—Cd1—N2A—C15A	30.95 (18)	N1—C6—C1—S1	0.5 (3)
O4A—Cd1—N2A—C15A	−114.2 (2)	C7—S1—C1—C2	179.9 (3)
N2—Cd1—N2A—C15A	−50.6 (3)	C7—S1—C1—C6	−0.3 (2)
O6—Cd1—N2A—C15A	−164.77 (18)	C7—N1—C8—C9	−106.3 (4)
O4—Cd1—N2A—C15A	−41.31 (19)	C6—N1—C8—C9	66.0 (4)
O5A—Cd1—N2A—C11A	−102.8 (2)	O2—C9—C8—N1	19.0 (5)
O5—Cd1—N2A—C11A	−158.1 (2)	O3—C9—C8—N1	−162.7 (3)
O6A—Cd1—N2A—C11A	151.1 (2)	C6—N1—C7—O1	−179.9 (3)
O4A—Cd1—N2A—C11A	5.9 (2)	C8—N1—C7—O1	−7.2 (5)
N2—Cd1—N2A—C11A	69.6 (3)	C6—N1—C7—S1	0.3 (3)
O6—Cd1—N2A—C11A	−44.6 (2)	C8—N1—C7—S1	173.0 (2)
O4—Cd1—N2A—C11A	78.8 (2)	C1—S1—C7—O1	−179.8 (3)
O5A—Cd1—N2A—C13A	17.12 (19)	C1—S1—C7—N1	0.0 (2)
O5—Cd1—N2A—C13A	−38.2 (2)	N1—C6—C5—C4	178.5 (3)
O6A—Cd1—N2A—C13A	−89.0 (2)	C1—C6—C5—C4	−1.3 (5)
O4A—Cd1—N2A—C13A	125.8 (2)	C6—C5—C4—C3	1.1 (6)
N2—Cd1—N2A—C13A	−170.6 (2)	C6—C1—C2—C3	1.3 (5)
O6—Cd1—N2A—C13A	75.2 (2)	S1—C1—C2—C3	−179.0 (3)
O4—Cd1—N2A—C13A	−161.29 (19)	C5—C4—C3—C2	0.4 (6)
O5A—Cd1—N2—C15	74.1 (2)	C1—C2—C3—C4	−1.6 (6)
O5—Cd1—N2—C15	123.6 (2)	C7A—N1A—C6A—C5A	179.6 (3)
O6A—Cd1—N2—C15	−170.6 (2)	C8A—N1A—C6A—C5A	1.5 (5)
N2A—Cd1—N2—C15	−96.4 (3)	C7A—N1A—C6A—C1A	−1.0 (4)
O4A—Cd1—N2—C15	−37.6 (2)	C8A—N1A—C6A—C1A	−179.1 (3)
O6—Cd1—N2—C15	27.5 (2)	C6A—N1A—C7A—O1A	−179.2 (4)
O4—Cd1—N2—C15	−106.2 (2)	C8A—N1A—C7A—O1A	−1.1 (6)
O5A—Cd1—N2—C11	−166.3 (2)	C6A—N1A—C7A—S1A	1.1 (4)
O5—Cd1—N2—C11	−116.8 (2)	C8A—N1A—C7A—S1A	179.2 (2)
O6A—Cd1—N2—C11	−51.1 (2)	C1A—S1A—C7A—O1A	179.6 (4)
N2A—Cd1—N2—C11	23.2 (3)	C1A—S1A—C7A—N1A	−0.7 (3)
O4A—Cd1—N2—C11	81.9 (2)	C7A—N1A—C8A—C9A	111.3 (4)
O6—Cd1—N2—C11	147.1 (2)	C6A—N1A—C8A—C9A	−70.7 (4)
O4—Cd1—N2—C11	13.4 (2)	C3A—C2A—C1A—C6A	−0.2 (6)
O5A—Cd1—N2—C13	−47.0 (2)	C3A—C2A—C1A—S1A	−179.7 (3)
O5—Cd1—N2—C13	2.5 (2)	C5A—C6A—C1A—C2A	0.3 (5)
O6A—Cd1—N2—C13	68.3 (2)	N1A—C6A—C1A—C2A	−179.2 (3)
N2A—Cd1—N2—C13	142.5 (2)	C5A—C6A—C1A—S1A	179.9 (3)
O4A—Cd1—N2—C13	−158.7 (2)	N1A—C6A—C1A—S1A	0.4 (3)
O6—Cd1—N2—C13	−93.5 (2)	C7A—S1A—C1A—C2A	179.7 (4)
O4—Cd1—N2—C13	132.8 (2)	C7A—S1A—C1A—C6A	0.1 (3)
O5A—Cd1—O6—C14	−147.9 (3)	N1A—C6A—C5A—C4A	179.8 (3)
O5—Cd1—O6—C14	−72.6 (3)	C1A—C6A—C5A—C4A	0.4 (5)
O6A—Cd1—O6—C14	−79.0 (4)	N1A—C8A—C9A—O2A	−5.2 (5)
N2A—Cd1—O6—C14	156.2 (3)	N1A—C8A—C9A—O3A	173.6 (3)
O4A—Cd1—O6—C14	106.0 (3)	C1A—C2A—C3A—C4A	−0.6 (7)
N2—Cd1—O6—C14	−2.9 (3)	C2A—C3A—C4A—C5A	1.4 (7)
O4—Cd1—O6—C14	49.0 (3)	C6A—C5A—C4A—C3A	−1.3 (6)

### Hydrogen-bond geometry (Å, º)

**Table:**

*D*—H···*A*	*D*—H	H···*A*	*D*···*A*	*D*—H···*A*
O4—H4···O3^i^	0.87 (3)	1.82 (2)	2.676 (4)	170 (4)
O4*A*—H4*A*···O2*A*	0.87 (3)	1.91 (3)	2.748 (4)	161 (3)
O5—H5···O2	0.87 (3)	1.81 (3)	2.661 (3)	166 (3)
O5*A*—H5*A*···O3*A*^ii^	0.87 (2)	1.83 (3)	2.640 (4)	154 (2)
O6—H6···O3*A*^ii^	0.85 (2)	2.04 (2)	2.749 (5)	141 (3)
O6*A*—H6*A*···O3	0.86 (2)	1.79 (2)	2.640 (4)	172 (2)
C11*A*—H11*D*···O1^iii^	0.97	2.47	3.282 (6)	142

Symmetry codes: (i) −*x*+2, −*y*, −*z*+2; (ii) −*x*+1, −*y*+1, −*z*+2; (iii) −*x*+1, −*y*, −*z*+2.
